# Applications of three-dimensional printing in percutaneous closure of aortic-to-right ventricle fistula after transcatheter aortic valve replacement: a case report

**DOI:** 10.1093/ehjcr/ytae112

**Published:** 2024-04-03

**Authors:** Julio Echarte-Morales, Irene Toribio-García, Alfredo Redondo Diéguez, Armando Pérez de Prado, Felipe Fernández-Vázquez

**Affiliations:** Montefiore-Einstein Center for Heart and Vascular Care, Montefiore Medical Center, Albert Einstein College of Medicine, 111 E 210 St, Bronx, NY 10467, USA; Department of Cardiology, University Hospital of Leon, Altos de Nava s/n, 24008, Leon, Spain; Department of Cardiology, University Hospital of Leon, Altos de Nava s/n, 24008, Leon, Spain; Department of Cardiology, Hospital Clínico Universitario de Valladolid, CIBERCV, Av. Ramon y Cajal 3, 47003, Valladolid, Spain; Cardiology Department, University Hospital of Santiago, Rua de Choupana s/n, 15706, Santiago de Compostela, Spain; Department of Cardiology, University Hospital of Leon, Altos de Nava s/n, 24008, Leon, Spain; Department of Cardiology, University Hospital of Leon, Altos de Nava s/n, 24008, Leon, Spain

**Keywords:** 3D printing model, Aortic-to-right ventricle fistula, Transcatheter aortic valve replacement, TAVI, TAVR complication, Aortic stenosis, Case report, 4.2 Aortic stenosis, 6.4 Acute heart failure, 4.10 Prosthetic valves

## Abstract

**Background:**

Percutaneous closure of aortic-to-right ventricle (ARV) fistula has emerged as an alternative to surgical management in selected cases. The use of three-dimensional (3D) printing in interventional planning for structural heart disease provides a concrete understanding, and it is useful in diagnostic assessment and to guide treatment approaches and to simulate procedures.

**Case summary:**

We report a case of a 70-year-old male presenting in cardiogenic shock due to severe aortic stenosis and reduced left ventricular ejection fraction. The patient had several comorbidities and was deemed not eligible for cardiac surgery. After transcatheter aortic valve replacement (TAVR), an ARV fistula was discovered on the TTE. Due to complex anatomical considerations, a 3D printed model of the patient’s anatomy was employed to supplement the decision-making process and technical planning of percutaneous ARV closure. Successful closure of the fistula with the use of the Amplatzer atrial septal occluder was subsequently performed.

**Discussion:**

Three-dimensional printing improves the understanding of complex structures of cardiac diseases, allowing for enhanced planning and simulation of the procedure. This case, demonstrating the effective percutaneous closure of a TAVR-related ARV fistula facilitated by the use of 3D printed anatomical models in the pre-procedural phase, highlights the technology’s potential in advancing patient-specific treatment approaches.

**Learning points**

- Three-dimensional printing proves to be an invaluable aid in planning the procedure and determining the best treatment approach.
- The formation of an aortic-to-right ventricular fistula is a significant yet uncommon complication following transcatheter aortic valve replacement.
- Closing this fistula is technically demanding because of the intricate anatomy involved.

## Introduction

Transcatheter aortic valve replacement (TAVR) is an established procedure to treat aortic stenosis (AS) in patients irrespective to their surgical risk.^1,2^ Aortic-to-right ventricle (ARV) fistula is a rare complication after TAVR, and it is associated with high mortality, and treatment presents a technical challenge.^3^ In this study, we describe percutaneous closure of an ARV fistula using three-dimensional (3D) printing to enhance and support pre-procedure planning.

## Summary figure

**Table:** 

Time	Salient event
**Day 0**	The patient presents with cardiogenic shock due to severe aortic stenosis and reduced left ventricular ejection fraction.
**Day 1**	Left heart catheterization evidencing critical stenosis of proximal left artery descending (LAD) and aortic valve invasive mean gradient of 50 mmHg.
**Day 2**	Patient underwent percutaneous coronary intervention of LAD and transcatheter aortic valve replacement.
**Day 5**	The patient was discharged from the hospital.
**Day 60**	The patient’s functional class worsened, and the heart team decided to close the aortic-to-right ventricular fistula. A computed tomography scan was planned with subsequent three-dimensional modelling of the patient’s anatomy to facilitate procedural planning.
**Day 99**	The patient underwent successful aortic-to-right ventricular fistula closure with an 18 mm Amplatzer atrial septal occluder occluder.

## Case presentation

A 70-year-old man with a prior medical history of hypertension, hyperlipidaemia, smoking, and severe peripheral arterial disease treated with femoro-femoral bypass presented to the emergency department with hypotension and severe dyspnoea. On physical examination, he had a slow rising pulse and auscultation revealed a 3/6 ejection systolic murmur radiating to the carotids, with reduced second heart sound. Presenting vital signs included 85/60 mmHg, 120 b.p.m., 28 breaths/min, and oxygen saturation of 80% on room air. Transthoracic echocardiography (TTE) showed reduced left ventricular ejection fraction (30%) and low-flow, low-gradient AS (pressure gradients of 55 mmHg peak/34 mmHg mean; peak velocity of 3.5 m/s; stroke volume index 28.6 mL/m^2^; and aortic valve area of 0.59 cm^2^). Mechanical ventilation and inotropes were initiated. Admission labs were notable for elevated N-terminal pro B-type natriuretic peptide (NT-proBNP) (14 960 pg/mL) and high-sensitivity cardiac troponin T (200 ng/L). Electrocardiogram showed a right bundle branch block and normal PR segment. A left heart catheterization was performed 24 h after admission, showing a critical stenosis in proximal left artery descending (LAD). Severity assessment of aortic stenosis was completed invasively (aortic valve peak to peak pressure gradient of 63 mmHg and mean gradient of 50 mmHg).

Based on his age, comorbidities, and the haemodynamic presentation, the heart team decided to perform proximal LAD stenting followed by TAVR. A multi-slice cardiac computed tomography (CT) showed an aortic valve annulus diameter of 29.7 mm and a perimeter of 94.4 mm. Of relevance, a significant left ventricular outflow tract calcium nodule was also noted. In addition, obstruction of the right external iliac artery and femoro-femoral bypass occluded with obstruction of the superficial femoral artery were observed.

**Figure 1:**
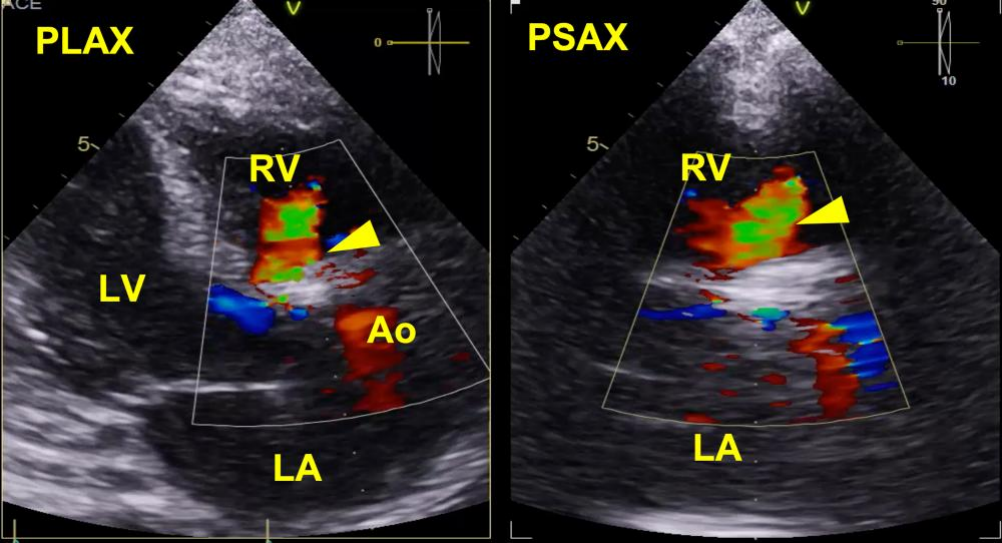
Transthoracic echocardiogram with colour flow Doppler evidencing an aortic-to-right ventricular fistula (arrowhead) status post-TAVR. Ao, aortic root; LA, left atrium; LV, left ventricle; PLAX, parasternal long axis; PSAX, parasternal short axis; RV, right ventricle.

Angioplasty with drug-eluting stent (XIENCE 3 × 28 mm) was performed on the proximal LAD. A 25 mm balloon valvuloplasty (True Balloon) was performed, and a balloon-expandable (Edwards SAPIEN 3) 29 mm valve was implanted via left transfemoral route. The post-procedural TTE revealed high-velocity left-to-right systolic–diastolic flow from the aortic root (immediately above the prosthesis at the level of the right coronary sinus) to the right ventricle suggestive of a fistula with a calculated Qp/Qs > 2 (*Figure 1*). During the hospitalization, serial TTEs were performed for monitoring, with stability of the ARV fistula and right ventricular function. The patient was discharged, but during follow-up, the functional class worsened with persistent high levels of NT-proBNP (>2000 pg/mL) despite optimal medical treatment. The heart team decided to close the ARV fistula percutaneously 2 months after TAVR.

**Figure 2:**
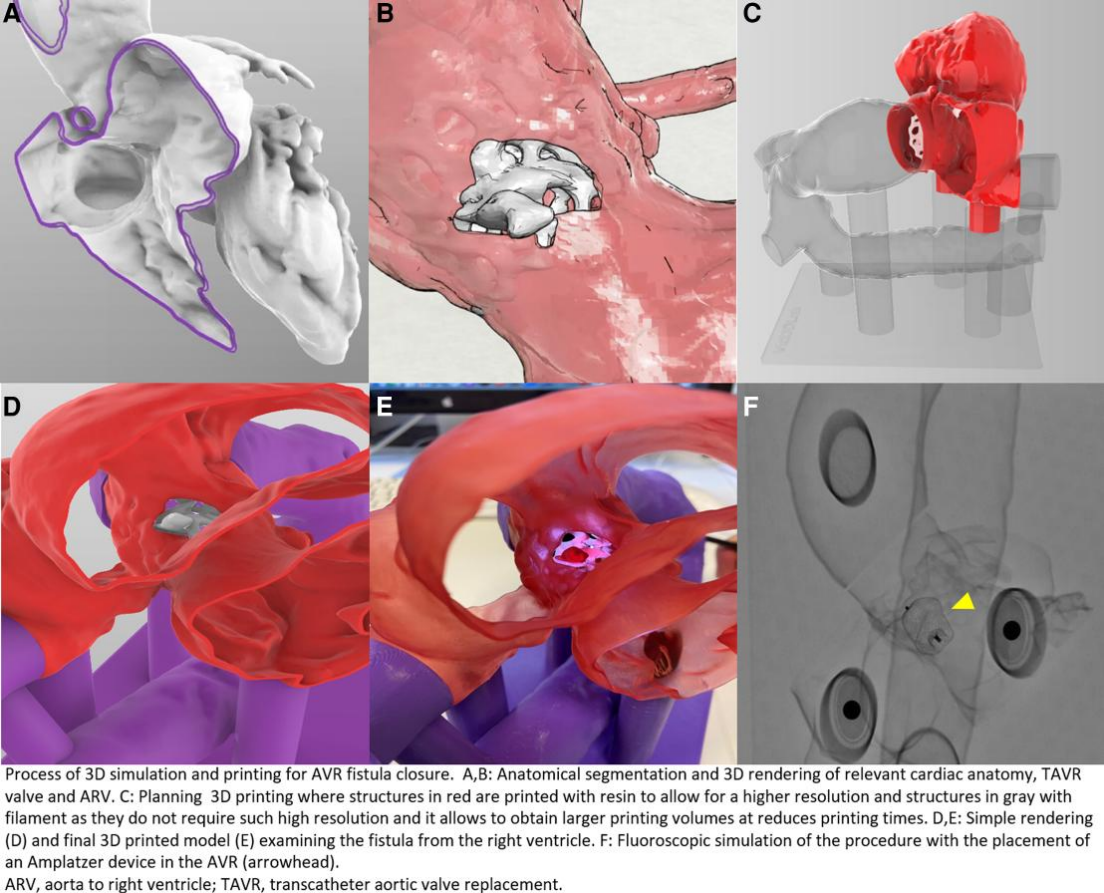
Generation of three-dimensional (3D) biomodel of aortic-to-right ventricular fistula. (*A* to *B*) Three-dimensional rendering evidencing the location of the ARV fistula, TAVR valve, and relevant cardiac anatomy. (*C* to *D*) Three-dimensional computer model used for 3D printing. (*E* to *F*) Three-dimensional printed model and physical procedure simulation with the placement of an Amplatzer occluder in the ARV fistula (arrowhead). ARV, aorta to right ventricle; TAVR, transcatheter aortic valve replacement; ARV, aorta to right ventricle; TAVR, transcatheter aortic valve replacement.

Due to the complex anatomy and technical difficulty of the procedure, it was decided to create a 3D model of the heart to better plan the procedure (*Figure 2*). The patient’s CT scan was segmented using Mimics software (Materialise, Belgium), generating a high-fidelity, patient-specific model. To allow for physical inspection and benchtop testing by the interventional team, the 3D biomodel was then transferred to a prototyping program (Rhinoceros software 5.0, Robert McNeel & Associates, Seattle, WA, USA) to plan the 3D printing process and subsequently print the model using a combination of resin and filament 3D printers. Simulations were exclusively performed on this 3D printed model to better understand the complex anatomy and determine the feasibility of intervention (see Supplementary material online). The simulation process was performed under fluoroscopy, simulating either an antegrade or retrograde approach. The deployment of the Amplatzer device was also simulated step by step.

**Figure 3:**
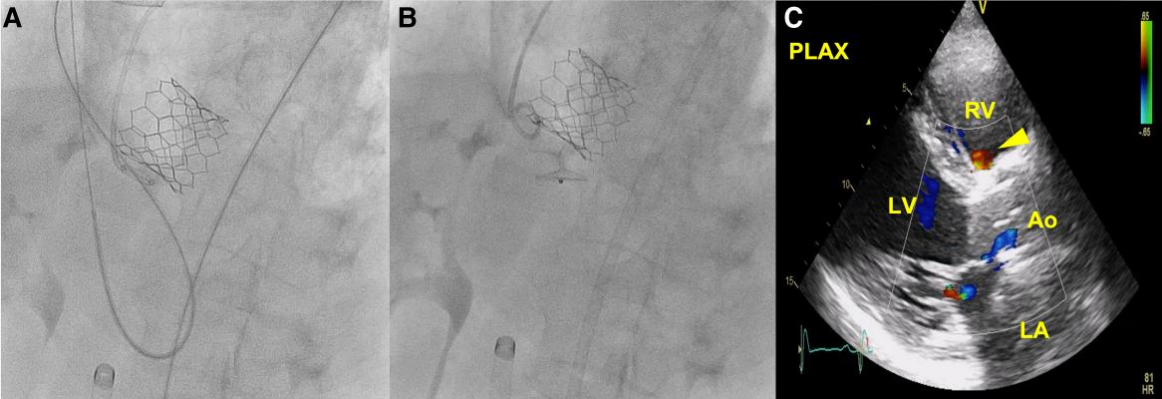
Percutaneous closure of the ARV fistula. (*A* and *B*) Deployment of an 18 mm Amplatzer atrial septal occluder into ARV fistula. (*C*) TTE showing the reduction of the shunt from the aorta root to the right ventricle after Amplatzer deployment (arrowhead). Ao, aortic root; ARV, aorta to right ventricle; LA, left atrium; LV, left ventricle; RV, right ventricle; TTE, transthoracic echocardiogram.

An antegrade approach (through the right femoral vein) was attempted crossing the defect with a 0.035 in hydrophilic wire with a straight tip (Terumo Radifocus®), and an arteriovenous loop was established using a 20 mm Goose Neck snare (Palex Medical). A 9F Amplatzer® TorqVue® (St. Jude Medical) delivery sheath was then advanced from the right ventricle, crossing the defect and entering the aorta. This approach was unsuccessful because the VSD Amplatzer® device consistently migrated towards the RV and did not secure well between the sinus of Valsalva and the TAVR. Therefore, a retrograde approach was perofrmed, and an 18 mm Amplatzer® atrial septal occluder occluder was implanted. The post-procedural TTE showed a decreased residual fistula without significant shunt and a calculated Qp/Qs 1.5 (*Figure 3*). At 2 years follow-up, the patient was stable, with improvement in functional class [New York Heart Association (NYHA) I–II/IV] and NT-proBNP levels lower than 1000 pg/mL.

## Discussion

Aortic-to-right ventricle fistula can be due to congenital diseases, infective endocarditis, and after cardiac surgical procedures as interventricular septal defect reparation or Ross technique. Factors such as the use of balloon-expandable valves, the depth of the prosthesis, larger sized devices, and excessive post-dilatation have been linked to an increased risk of aortic annulus rupture or fistula formation during TAVR.^3^ While there is no agreement on the optimal timing for intervening in these cases, if there is a notable shunt with high pulmonary pressures, indications of substantial right ventricular dysfunction, or symptoms such as worsening of functional class with increase in cardiac biomarkers, closure should be performed.

There are some cases of ARV fistula closure published in the literature using multimodality imaging with good outcomes.^4–6^ The most common imaging techniques used were transoesophageal echocardiogram, CT, and intracardiac echocardiography. In addition to these imaging techniques, computer simulation plays a crucial role in pre-procedural planning, especially in complex cases like ours. These techniques enable the virtual simulation of procedures using patient-specific anatomical data, offering insights into possible outcomes and procedural challenges.^7^ In our case, computer simulations provided additional perspectives on the intervention’s feasibility and helped in fine-tuning the approach for the percutaneous closure of the ARV fistula. So far, this is the first case report of ARV fistula closure assisted by using a 3D printed model for pre-procedural planning. Obtaining a 3D printed model, that is to say a 3D representation of an organ or tissue of interest, is a unique pathway that opens multiple possibilities to apply relatively novel image representation and simulation technologies in the field of medicine and that has the potential to provide added value in the diagnosis and treatment of various pathologies.^8,9^ The modality of medical images for obtaining biomodels can be diverse: CT, nuclear magnetic resonance, 3D ultrasound, and positron emission tomography.

There are different types of 3D printing depending on the physical phenomenon that allows the aggregation of new layers of material. The most widely used technologies are fused deposition modelling and photopolymerization. Other technologies include selective laser sintering or printing using binder jetting (jetting or microjetting).

In our case, the patient’s biomodel was generated from a post-TAVR CT scan and printed using a combination of resin and filament materials, depending on the anatomic structure. Resin was used for the region of the ARV fistula, left atrium, and right and left ventricles due to its superior resolution. However, the aorta and vena cava were printed using a filament material since they did not require such high resolution and it allowed for larger printing volumes and reduced printing times. Both virtual and physical models were critical in deeming the intervention feasible and guiding the successful percutaneous implantation of an Amplatzer plug.

Despite their benefits, patient-specific computer modelling and 3D printed models have limitations. The materials used for 3D printing may not fully mimic human tissue properties, which can impact procedural accuracy. Additionally, the cost and time required for producing these models can be significant. Computer modelling and simulation accuracy is also dependent on the quality of input data, with any inaccuracies potentially affecting simulation reliability.

## Conclusion

In conclusion, this cases report demonstrates that percutaneous closure of ARV fistula is a feasible alternative in patients with high surgical risk. Three-dimensional printing improves the understanding of complex structures of cardiac diseases, allowing the planning and simulation of the procedure, thus achieving an effective therapeutic approach.

## Supplementary Material

ytae112_Supplementary_Data

## Data Availability

The data underlying this article will be shared on reasonable request to the corresponding author.

## Lead author biography

Julio Echarte-Morales was born in Havana, Cuba. He graduated in medicine in 2015 from Universidad de Ciencias Medicas de La Habana. He completed his general cardiology fellowship in Complejo Asistencial Universitario de Leon, Spain. Currently, he is a research fellow at Montefiore Medical Center, USA.

## Supplementary material

**Consent:** The authors declare that consent has been given for the publication of this detailed case study in accordance with the Committee on Publication Ethics (COPE) guidelines.

**Funding:** Dr Echarte-Morales was supported by Sociedad Española de Cardiología through a mobility grant (SEC/PRS-MOV-INT 22/001) and is an associate editor of *European Heart Journal – Case Reports*.
