# Supplementary material for: Applications of three-dimensional printing in percutaneous closure of aortic-to-right ventricle fistula after transcatheter aortic valve replacement: a case report
Source: Eur Heart J Case Rep. 2024 Apr 3;8(5):ytae112. doi: 10.1093/ehjcr/ytae112 (PMC11068068; doi:10.1093/ehjcr/ytae112)
Supplement: ytae112_Supplementary_Data [file ytae112_supplementary_data.zip › 23-00779_Authorship Modification.pdf]

February 13th, 2024

Dear Editorial Office Team,

We are writing to inform you that all authors of the manuscript titled ``Applications of 3D printing in percutaneous closure of aortic to right ventricle fistula after transcatheter aortic valve replacement: A case report´´ are fully aware of the change in authorship. After careful consideration and discussion among all involved parties, we are collectively in agreement to proceed with publication under the revised authorship.

Furthermore, we would like to confirm that all authors, including the individual whose name has been removed, have signed this letter to signify their understanding and acceptance of the change. We appreciate your attention to this matter and look forward to the publication of our work.

Sincerely,

Julio Echarte Morales

Matteo Sturla

Irene Toribio García

Alfredo Redondo Diéguez

Armando Pérez de Prado

Felipe Fernández Vázquez
